# Metabolism and Transcription in Cancer: Merging Two Classic Tales

**DOI:** 10.3389/fcell.2017.00119

**Published:** 2018-01-05

**Authors:** Natalia Martín-Martín, Arkaitz Carracedo, Verónica Torrano

**Affiliations:** ^1^CIC bioGUNE, Bizkaia Technology Park, Derio, Spain; ^2^Centro de Investigación Biomédica en Red Cáncer, Madrid, Spain; ^3^IKERBASQUE, Basque Foundation for Science, Bilbao, Spain; ^4^Biochemistry and Molecular Biology Department, University of the Basque Country (UPV/EHU), Bilbao, Spain

**Keywords:** cancer metabolism, nutrient sensing networks, transcription factors, histone acetylation, DNA and histone methylation, gene expression regulation

## Abstract

Cellular plasticity, or the ability of a cancer cell to adapt to changes in the microenvironment, is a major determinant of cell survival and functionality that require the coordination of transcriptional programs with signaling and metabolic pathways. In this scenario, these pathways sense and integrate nutrient signals for the induction of coordinated gene expression programs in cancer. This minireview focuses on recent advances that shed light on the bidirectional relationship between metabolism and gene transcription, and their biological outcomes in cancer. Specifically, we will discuss how metabolic changes occurring in cancer cells impact on gene expression, both at the level of the epigenetic landscape and transcription factor regulation.

## Introduction

The advances toward curative treatments for cancer are nowadays based on three pillars of research: (i) early detection, (ii) molecular stratification of high-risk patients and (iii) the selection of the most appropriate therapeutic strategy. New insights in the molecular understanding of cancer has led to a paradigmatic change in the way we combat the disease, introducing the concept of precision medicine: patient's stratification and personalized therapy.

**Figure 1:**
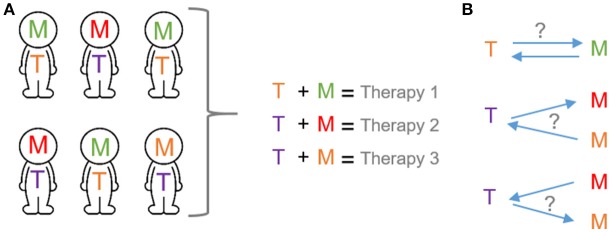
Transcription and metabolic programs as potential tool for precision medicine. **(A)** The application of the precision medicine concept will depend on the selection of specific cancer therapies based on both transcriptional and metabolic programs of cancer patients. **(B)** Mechanistic basis of precision medicine. Bidirectional interplay between transcription and metabolic programs. T, transcription programs; M, metabolic programs. Each color exemplifies different programs.

In the recent years there has been a renaissance in the study of the cross-interaction between two important “usual suspects” in cancer: gene expression and metabolism (Hanahan and Weinberg, 2011). Both research areas have inherited potential to be applied to precision medicine. On the one hand, the study of transcriptional regulators can potentially lead to the development of stratification tools. On the other, the stratification can define which cancer patients will benefit from a given metabolic-based therapeutic approach (Figure 1A).

Along the process of transformation, the acquisition of pro-survival abilities is a crucial determinant that enables cancer cells to adapt to the ever-changing environment (Hanahan and Weinberg, 2011). This master adaptation is based, in part, on the connection between nutrient sensing and gene expression programs. As a consequence, cancer cells rewire their metabolism to activate the fittest metabolic rate for cancer homeostasis. This type of response requires a circuit in which cellular metabolism and gene transcription must be bidirectionally connected and tightly coordinated (Figure 1B).

One of the most important cellular regulatory mechanism that determine which genes are activated is the packing of DNA and histones in chromatin or epigenetic remodeling. Post-translational modifications of histones and DNA—mainly acetylation and methylation—alter the structure of chromatin, helping or preventing the recruitment of transcription factors complexes that will ultimately regulate gene expression. At the same time, changes in gene expression in response to environmental fluctuations are led by post-translational modifications or activation of transcription factors. Metabolism is the process of energy transduction that encompasses a network of chemical reactions tightly regulated by environmental changes. The idea that epigenetics and gene transcription can be influenced by products of metabolic pathways was proposed many years ago (Shi and Shi, 2004), but the biological relevance of this concept in tumorigenic processes has remained largely unknown.

Systematic profiling of cancer specimens has determined the existence of epigenetic alterations across the genome that potentially regulate gene expression and are associated with tumor progression (Baylin and Jones, 2011). This expanding field is coming together with cancer metabolism. During transformation, the entire metabolic network is rewired to efficiently convert nutrients to biosynthetic precursors to sustain cancer cell growth (Hanahan and Weinberg, 2011).

**Figure 2:**
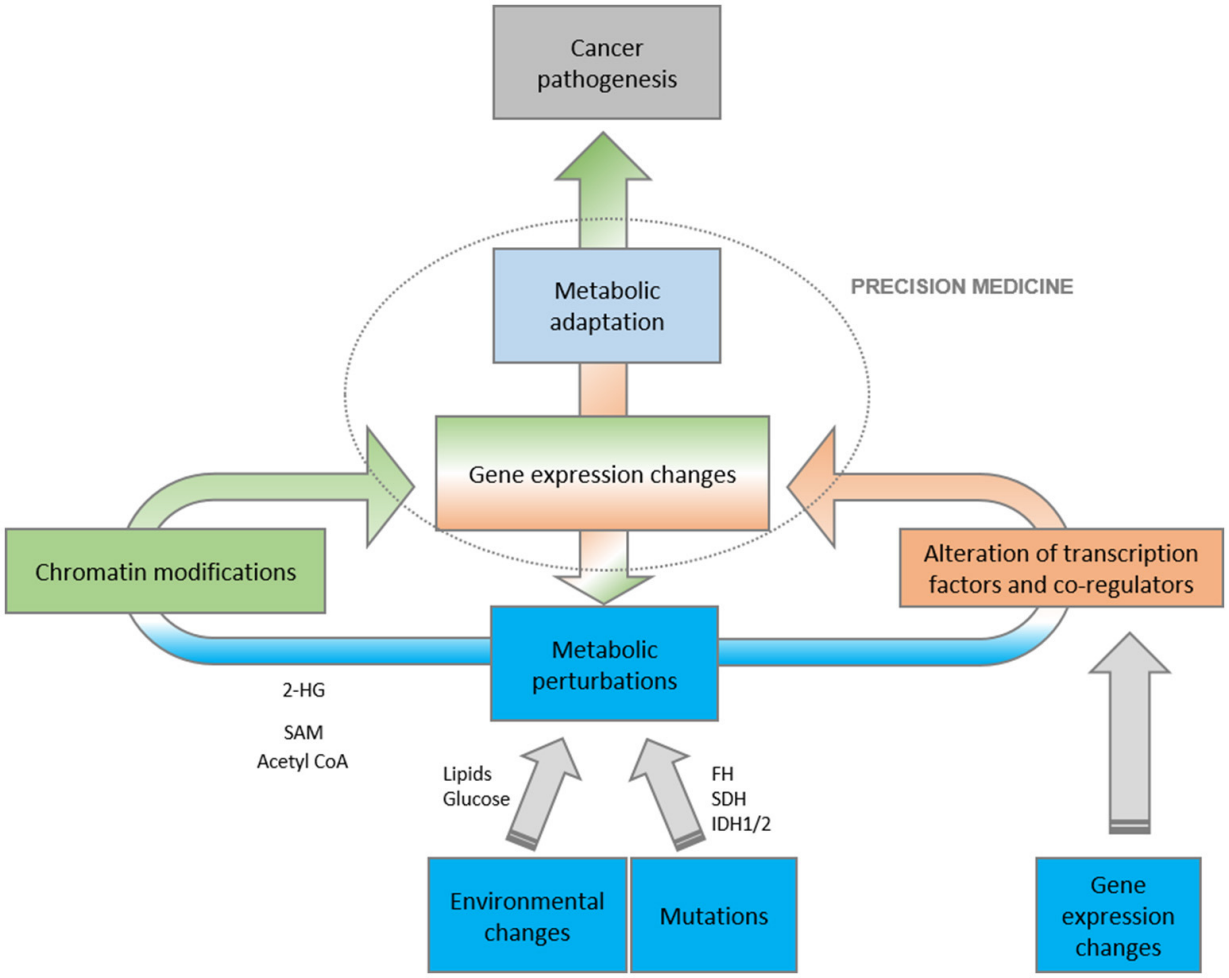
Schematic representation of the complex relationship between metabolism and gene expression. Metabolic perturbations, as a result of environmental, mutational and metabolic insults, directly impact on gene expression programs, both at the level of epigenetic changes and transcriptional activities. The final outcome is that the conjunction of metabolism and transcription have a profound impact on oncogenesis.

Metabolic and epigenetic enzymes are frequently components of the same tumorigenic pathway. Thus, metabolic rewiring occurring in cancer can impact on the regulation of chromatin structure and, therefore, cancer-related gene expression. Conversely, nutrient availability, or extracellular signals within the tumor microenvironment can fine-tune the expression of metabolic genes through epigenetic modifications and transcriptional regulation (Figure 2).

## Impact of metabolism and its products in gene expression programs

Most chromatin-modifying enzymes use co-factors and substrates that are critical metabolites of the intermediary metabolism. The availability of these metabolites can influence the capacity of the cell to write or erase chromatin marks, highlighting the intimate link between the metabolic state, epigenetic regulation and gene expression.

### DNA and histone methylation

In human DNA, cytosines are typically methylated at CpG islands located in promoter regions and associated with transcriptional regulation. Cancers frequently display global DNA hypomethylation but hypermethylation of CpG islands in genomic regions where tumor suppressor genes are located (Hansen et al., 2011). These histone methyl marks can either activate or repress gene expression (Kinnaird et al., 2016).

Methylation is linked to the intermediary metabolism through S-adenosyl methionine (SAM), the primary source of methyl groups that is generated in the folate and methionine cycles (Maddocks et al., 2016; Mentch and Locasale, 2016). The activities of both histone methyltransferases (HMT) and DNA methyltransferases (DNMT) depend on the levels of intracellular SAM which varies based on serine and methionine availability. The deprivation of these essential amino acids induce reversible and rapid changes in histone and DNA methylation, which in turn change the transcriptional landscape of cancer cells (Mentch et al., 2015; Maddocks et al., 2016). In the light of these data, the methionine cycle and the sensing of SAM availability provide a direct link between intermediary metabolism and chromatin state in cells.

Interestingly, system biology approaches have revealed methionine cycle and one-carbon metabolism gene networks as major determinants of DNA methylation status in human cancer and cancer survival predictors (Mehrmohamadi et al., 2016). Indeed, dysregulation of histone methylation in specific chromatin regions is a major selective force for tumor progression and metastatic potential (McDonald et al., 2017). Of note, the epigenetic changes associated with distant metastasis are strongly dependent on the oxidative branch of the pentose phosphate pathway (oxPPP). This dependency confers selective advantages to the disseminated cells enabling their metastatic spread. In distal metastasis sites, oxPPP is coupled to epigenetic programs that promote tumorigenesis (McDonald et al., 2017).

The demethylation reaction is also susceptible to metabolic fluctuations. The enzymatic removal of methyl groups is regulated by histone and DNA demethylases whose activities are modulated by the tricarboxylic acid (TCA) cycle intermediates alpha-ketoglutarate (α-KG), fumarate and succinate. When presented in sufficient concentration, α-KG acts as a positive co-factor of the demethylase activity, while fumarate and succinate are competitive inhibitors of multiple histone demethylases (Xiao et al., 2012). The activity of these enzymes can be dramatically altered by mutations in key metabolic enzymes. Inactivating mutations affecting the mitochondrial succinate dehydrogenase (SDH) complex subunits and fumarate hydratase (FH) are driver mutations in a subset of cancers (Tomlinson et al., 2002; Janeway et al., 2011; Pantaleo et al., 2011; Castro-Vega et al., 2014; Clark et al., 2014). These mutations lead to the accumulation of succinate and fumarate and the subsequent inhibition of α -KG-dependent dioxygenases (Xiao et al., 2012). The direct contribution of fumarate accumulation and epigenetics to tumorigenesis has been elegantly shown in the context of *FH* loss. In this scenario, fumarate accumulation elicits epigenetic changes in a regulatory region of the antimetastatic miRNA cluster *mir-200ba429*. In turn, the suppression of miR-200 leads to the expression of epithelial-to-mesenchymal-transition (EMT)-related transcription factors and the enhancement of migratory properties (Sciacovelli et al., 2016). Deficiency of SDH is associated with global DNA methylation changes (Killian et al., 2013) and the downregulation of neuroendocrine differentiation genes linked to a migratory phenotype (Letouze et al., 2013).

Upstream of SDH in the TCA cycle, isocitrate dehydrogenase (IDH) catalyzes the oxidative decarboxylation of isocitrate, producing α-KG and CO_2_. IDH genes are the most frequently mutated metabolic genes in cancers driving global epigenetic changes (Figueroa et al., 2010; Ward et al., 2010; Cairns et al., 2012). Mutations in IDH1/2 have oncogenic properties and impede the synthesis of α-KG but favor the formation of the oncometabolite 2-hydroxyglutarate (2-HG) (Dang et al., 2009; Ye et al., 2013). In turn, 2-HG accumulation inhibits DNA demethylation (Losman et al., 2013) and primes cancer cells for transformation (Figueroa et al., 2010; Lu et al., 2012; Turcan et al., 2012). However, the production of 2-HG is not restricted to an IDH mutated background. For example, in hypoxia wild-type IDH2 produces 2-HG as a by-product (Wise et al., 2011). In ER-negative breast cancer patients, the accumulation of 2-HG define a subgroup of wild-type IDH2 patients with specific hypermethylation phenotype and poor clinical outcome (Terunuma et al., 2014). This work suggests that the metabolic-epigenetic axis could be reflected in tumor subtypes of clinical relevance.

Beyond cancer biology, but conceptually connected, 2-HG has been proposed to act as an immunometabolite that links the environmental context to immune fate and function through a metabolic–epigenetic axis (Tyrakis et al., 2016; Xu et al., 2017). Given the important role of the immune system in the maintenance of chronic inflammation during tumorigenic processes (Numasaki et al., 2003; Grivennikov et al., 2012), these results may have implications for tumor immunology.

In summary, the accumulation of succinate, fumarate, and 2-HG contribute to cancer progression and position the Krebs cycle as mitochondrial custodian of the methylome (Figure 2).

### Histone acetylation

Global levels of nuclear histone acetylation are sensitive to overall acetyl CoA levels. Acetyl CoA is a key intermediate of central metabolism, which not only fuels ATP production via the TCA cycle, but also functions as an essential building block for the synthesis of fatty acids and sterols, and importantly histone acetylation. Acetyl CoA is generated from catabolic pathways of intermediary metabolism and at the same time used by anabolic processes such as lipid synthesis. In mammalian cells, there are three major enzymes that generate acetyl CoA: acetate-dependent acetyl-CoA synthetase 2 (ACSS2), citrate-dependent ATP-citrate lyase (ACLY) and mitochondrial pyruvate dehydrogenase complex (PDC). The relative importance of ACSS2, ACLY and PDC for nuclear histone acetylation differs on the basis of the developmental state, disease, tissue type and even subcellular location.

ACLY is the primary enzyme responsible for the synthesis of acetyl CoA from glucose-derived citrate and connects oncogenic signals to histone acetylation (Wellen et al., 2009; Lee et al., 2014). In the absence of ACLY, under nutrient deprivation or stress conditions, cells upregulate ACSS2, enabling cancer cells to utilize acetate to sustain tumor growth (Comerford et al., 2014; Mashimo et al., 2014; Schug et al., 2015) by providing acetyl CoA for fatty acid and phospholipid synthesis and histone acetylation (Zhao et al., 2016). In addition, under hypoxic conditions, acetate mediates epigenetic changes that specifically activate a lipogenic program and promote cancer cell survival (Gao et al., 2016). Importantly, ACSS2 has been recently identified as a chromatin-bound factor that regulates and coordinates gene expression programs related to long-term spatial memory (Mews et al., 2017). This is the first evidence of the direct and causal contribution of ACSS2-derived acetyl CoA to epigenetic modulation and gene expression.

Lipid-derived carbons are also a bona fide physiological source of acetyl CoA for histone acetylation. The acetyl CoA produced via the activation of fatty acid oxidation (FAO) is selectively used by histone acetyl transferases located at gene locus where key lymphatic and lipid-specific genes reside (McDonnell et al., 2016; Wong et al., 2017). These studies expand the landscape of nutrient sensing and uncover how lipids and metabolism are integrated by epigenetic events that control gene expression. In a cancer scenario, the uptake of fatty acids—mediated by CD36—and their oxidation sustain cancer-initiating cells and promote metastasis. Interestingly, these metastasis-initiating cells with high expression of CD36 are defined by a lipid metabolism transcriptional signature (Pascual et al., 2017). Although no link with epigenetic changes have been associated with this phenotype, we could predict that lipid uptake, and presumably its oxidation could play and important role in cell survival and cancer progression by regulating the epigenetic and transcriptional landscapes.

Due to its biochemical properties, the biosynthesis of acetyl CoA is thought to occur in the subcellular compartment where it is required. Therefore, the localized production of acetyl CoA by spatial regulation of its enzymatic producers would confer a high degree of specificity to metabolic regulation of histone acetylation and gene expression.

In the mitochondria, acetyl CoA is the main product of FAO, branch chain amino acid catabolism and pyruvate oxidation through the activity of PDC. Although PDC has classically been localized to the mitochondria, under metabolic insults, functional PDC translocate to the nucleus. There, it generates a nuclear pool of acetyl CoA that increases the acetylation of core histones important for S phase entry (Sutendra et al., 2014). In line, spatial regulation of ACSS2 confers specificity to the metabolic regulation of histone acetylation and together with ACLY were found in the nucleus (Takahashi et al., 2006; Wellen et al., 2009). Importantly, the “on site” generation of ACSS2-derived acetyl CoA at specific chromatin domains favors histone acetylation of key genes involved in long-term spatial memory, autophagy, cell survival and tumorigenesis (Bulusu et al., 2017; Li et al., 2017a; Mews et al., 2017).

Interestingly, the modulation of the mitochondrial protein VDAC1 induced a coordinated cascade of changes in mitochondrial metabolites that elicited a global metabolic re-programming in glioblastoma cells. This metabolic rewiring led to the activation of neural cell differentiation transcriptional programs and reversal oncogenic properties of glioblastoma cells (Arif et al., 2017).

In summary, chromatin-associated enzymes sense intermediary metabolism products and process this information into dynamic chromatin modifications that will ultimately regulate adaptive transcriptional programs associated with oncogenic processes.

## Transcriptional regulation of metabolic programs

The metabolic switch in cancer encloses a plethora of discrete enzymatic activities that must be coordinately altered in order to ensure the adaptation of cancer cells to environmental alterations (Loo et al., 2015). In the recent years, numerous reports have provided evidences of the cues regulating one or few enzymes within a metabolic pathway in cancer. However, the means of coordinated regulation of complex metabolic networks is starting to be elucidated (Torrano et al., 2016; Valcarcel-Jimenez et al., 2017).

Nutrients perturbations can be sensed directly by master transcriptional regulators of metabolism that will ultimately elicit the coordinated expression of genes required for metabolic adaptation in cancer cells (Figure 2). These programs allow the rapid adaptation to new biological states or external insults, and their contribution to cancer pathogenesis and progression has begun to emerge (Mouchiroud et al., 2014). More than fifty years ago an association between lipid metabolism and tumor progression was reported (Weinhouse et al., 1951) and since that time, the involvement of lipid metabolism in tumorigenesis has been thoroughly investigated.

Peroxisome-proliferator-activated receptors (PPARs), PPAR-α, PPAR-δ (also known as PPAR-β) and PPAR-γ, are members of the nuclear receptor superfamily of transcription factors that control lipid sensing and the transcriptional regulation of metabolic pathways (Michalik et al., 2006). PPARs regulate gene expression programs that impact on proliferation, differentiation and survival, thus controlling carcinogenesis in various tissues including liver, breast, lung, colon and bone marrow. The role of these nuclear factors in transformation has been controversial during the past years, being described as either tumor suppressor or oncogenes (Carracedo et al., 2012; Ito et al., 2012; Peters et al., 2015; Lakshmi et al., 2017; Martin-Martin et al., 2017; Sun et al., 2017). The activity of PPARs is modulated upon ligand binding and by a number of coactivator and corepressor proteins, the presence of which can stimulate or inhibit the transcriptional function of the receptor (Feige and Auwerx, 2007; Martin-Martin et al., 2017). One of the most studied co-regulators of PPARs function is PPAR gamma co-activator 1 alpha (PGC1α), a master transcriptional co-activator with broad functions in energy metabolism. Together, PPARs and PGC1α control mitochondrial function and FAO (Sugden et al., 2010) and have been implicated in the maintenance of hematopoietic stem cell pool, cancer survival and progression (Carracedo et al., 2012; Ito et al., 2012; Torrano et al., 2016; Valcarcel-Jimenez et al., 2017).

The classical nuclear receptors are known as the receptors for steroids such as estrogen, androgen, glucocorticoid, and progesterone, which are derivatives of cholesterol. Among these classical nuclear factors, the sterol regulatory element binding-proteins (SREBPs) are the master transcription factors that are highly sensitive to the intracellular levels of cholesterol. The cholesterol composition of cellular membranes is an essential metabolic requirement for cell division (Bengoechea-Alonso and Ericsson, 2016). Different cancer cell types adapt their metabolism to maintain high intracellular cholesterol levels through increased cholesterol uptake and the activation of lipogenic transcriptional programs dependent on SREBP-1 (Guo et al., 2011; Huang et al., 2012; Li et al., 2017b). These pathways converge into the accelerated endogenous production of cholesterol. It has been recently described the regulation of ACSS2 by SREBP in mammary epithelial cells, having this regulation an effect on fatty acid synthesis (Xu et al., 2018). Given the important role of ACSS2 as a central node between metabolism and epigenetic regulation in cancer, it is tempting to speculate that the cholesterol levels in cancer cells may have an impact on gene regulation through the modulation of ACSS2 enzymatic activity.

The transcriptional agonist properties of cholesterol are not limited to SREBPs. Cholesterol has been recently identified as a physiological and functional endogenous agonist of the estrogen-related receptor alpha (ERRα). Upon cholesterol binding, ERRα recruits PGC1α coactivators to DNA promoters and together serve as a critical metabolic sensors that regulate gene expression programs associated to osteogenesis, myogenesis and macrophage activation (Wei et al., 2016). This is the first evidence for cholesterol and the cholesterol biosynthetic pathway in the regulation of ERRα activity and biology.

Taken together, all these data position cholesterol as a master metabolite that control gene transcription programs via its interaction with nuclear factors.

ERRα and its transcriptional programs are implicated in metabolism and cancer progression. Increased ERRα activity is observed in melanoma, breast and ovarian cancer, colorectal carcinoma and osteosarcoma (Stein and McDonnell, 2006; Vazquez et al., 2013; Chen et al., 2014; Thewes et al., 2015). ERRs are nuclear receptors that exhibit ligand-dependent regulation, and their activity relies on the status of transcriptional co-activators and co-repressors (Feige and Auwerx, 2007). One such co-activators, PGC1α has been extensively studied in physiological conditions (Handschin, 2009). PGC1α controls transcriptional programs that increase the energetic yield (Scarpulla, 2011) and counteract oxidative stress (St-Pierre et al., 2006; Haq et al., 2013; Vazquez et al., 2013), which enables elevated oxidative mitochondrial activity (OXPHOS) coping with the accumulation of reactive oxidant species (ROS). PGC1α exerts paradoxical activities in different tumor types and biological conditions and recent studies highlight the importance of it in cancer metabolism (Vazquez et al., 2013; LeBleu et al., 2014; Sancho et al., 2015; Luo et al., 2016) and specifically through the regulation of ERRs (Haq et al., 2013; Vazquez et al., 2013; Torrano et al., 2016; Valcarcel-Jimenez et al., 2017).

The classical view of cancer metabolic wiring (Warburg effect) would predict that the PGC1α-ERRα axis and OXPHOS triggered are inherently tumor suppressive. However, recent studies uncover that factors such as mutational background, tissue or cell of origin and disease stage impose a pressure toward the best-adapted metabolic wiring during cancer progression. In melanoma and breast cancer, cells turn on PGC1α and their OXPHOS program, which impacts on cancer cell survival, proliferation and contribution to therapy resistance (Haq et al., 2013; Vazquez et al., 2013; LeBleu et al., 2014). Interestingly, during the process of metastasis, melanoma cells need to suppress PGC1α expression in order to regulate an adhesion and invasion transcriptional program (Luo et al., 2016). In line, OXPHOS PGC1α-induced metabolism represents a disadvantageous metabolic state in prostate cancer. Moreover, the decrease of PGC1α-ERRα transcriptional activity provides a selective advantage to metastasize and correlates with an increased disease recurrence (Torrano et al., 2016; Valcarcel-Jimenez et al., 2017).

These studies elegantly illustrate how the PGC1α-ERRα transcriptional axis can exert opposing activities in cancer progression, highlighting the metabolic diversity leading to metabolic adaptations during cancer progression in different cancer types.

## Concluding remarks

Metabolic rewiring and gene deregulation are both hallmarks of cancer (Hanahan and Weinberg, 2011) and are addictive for tumor cells (Bradner et al., 2017; Vander Heiden and DeBerardinis, 2017). Thus, the crosstalk between gene expression and metabolism are fundamental aspects of cellular adaptation to nutritional changes during tumorigenesis. An attractive approach to understand cancer and identify therapeutic targets is to discover the key components on which deregulated transcriptional and metabolic programs depend in cancer cells. We have outlined recent advances that described how coordinated gene expression programs are tightly and dynamically regulated by the metabolome, either at the level of chromatin modifications and transcription factor activities. In this scenario, metabolic alterations during cellular transformation drive aberrant gene expression which in turn will be key contributors to tumor development and progression. However, much remains to be discovered, and the study of the bidirectional contribution of metabolism to gene expression regulation will bring a more integrated understanding of cellular adaptations during cancer progression and, possibly new therapeutic opportunities.

## Author contributions

VT wrote the manuscript with essential contribution of AC and NM-M.

### Conflict of interest statement

The authors declare that the research was conducted in the absence of any commercial or financial relationships that could be construed as a potential conflict of interest.
